# Lowering latency and processing burden in computational imaging through dimensionality reduction of the sensing matrix

**DOI:** 10.1038/s41598-021-83021-6

**Published:** 2021-02-11

**Authors:** Thomas Fromentèze, Okan Yurduseven, Philipp del Hougne, David R. Smith

**Affiliations:** 1 University of Limoges, CNRS, XLIM, UMR 7252, 87000 Limoges, France; 2 Centre for Wireless Innovation (CWI), Institute of Electronics, Communications and Information Technology (ECIT), School of Electronics, Electrical Engineering and Computer Science (EEECS), Queen’s University Belfast, Belfast, BT3 9DT UK; 3 Univ Rennes, CNRS, IETR - UMR 6164, 35000 Rennes, France; 4 Department of Electrical and Computer Engineering, Center for Metamaterials and Integrated Plasmonics, Duke University, Durham, NC 27708 USA

**Keywords:** Imaging techniques, Electrical and electronic engineering, Imaging and sensing

## Abstract

Recent demonstrations have shown that frequency-diverse computational imaging systems can greatly simplify conventional architectures developed for imaging by transferring constraints into the digital layer. Here, in order to limit the latency and processing burden involved in image reconstruction, we propose to truncate insignificant principal components of the sensing matrix that links the measurements to the scene to be imaged. In contrast to recent work using principle component analysis to synthesize scene illuminations, our generic approach is fully unsupervised and is applied directly to the sensing matrix. We impose no restrictions on the type of imageable scene, no training data is required, and no actively reconfigurable radiating apertures are employed. This paper paves the way to the constitution of a new degree of freedom in image reconstructions, allowing one to place the performance emphasis either on image quality or latency and computational burden. The application of such relaxations will be essential for widespread deployment of computational microwave and millimeter wave imagers in scenarios such as security screening. We show in this specific context that it is possible to reduce both the processing time and memory consumption with a minor impact on the quality of the reconstructed images.

## Introduction

Imaging with microwaves brings about several advantages, such as all-weather operation, the use of non-ionizing radiation, the ability to see through most optically opaque materials and the availability of mature component technology. Due to these advantages, microwave imaging has proven to be useful in a wide range of applications ranging from security-screening^1–4^ to biomedical imaging^5–8^ and non-destructive testing^9–12^. Despite these advantages, conventional microwave imaging systems suffer from a number of challenges. In general, microwave imaging requires the synthesis of composite apertures to raster-scan the scene, either mechanically, such as in synthetic aperture radar^13–16^, or electronically, such as in phased arrays^17–21^. Mechanical scanning is not desirable in that data acquisition time can be significant, posing a major challenge for real-time operation. All-electronic operation can be achieved using phased arrays. However, beam synthesis using phased arrays requires that each antenna within the composite aperture has a phase-control circuit, or a phase-shifter, and preferably a power amplifier (to compensate for the insertion losses of the phase shifters). As a result, using phased arrays, all-electronic operation can require a large number of phase-shifting circuits and power amplifiers, significantly increasing the complexity and power-consumption of such apertures. Applications requiring high depth resolution will also be particularly constrained by the fractional bandwidth limitations of the active components of each transmission and reception chain.

To address these challenges, unusual modalities have been proposed that leverage ideas related to computational imaging^22–27^. Numerous proofs of concept of frequency-diverse computational imaging have recently been proposed^28–35^, where the scene information is encoded onto a set of measurement modes that exhibit quasi-randomness across the operating frequency band. In other words, the scene information is sampled on quasi-random bases by stepping through a number of frequency points across the operating frequency band (frequency sweep)^36^. Leveraging this quasi-random set of modes offered by engineered frequency-diverse systems eliminates the need for beam synthesis and requires a simple frequency sweep to achieve imaging. A three-dimensional (3D) microwave image of the scene can then be reconstructed by interacting the measurements of the scene with the transfer function of the frequency-diverse imaging system using computational reconstruction techniques, such as direct algorithms, including the matched-filter technique, or iterative algorithms, including the least-square technique^37^.

Using such frequency-diverse schemes for computational imaging of large scenes at microwave frequencies, such as in security-screening, can be a computationally demanding task. For a 3D scene discretised at the resolution limit of a finite-size aperture, the number of voxels to be reconstructed can be significant. Several techniques can be adopted to reduce the computational complexity of the imaging problem. For example, additionally using data from optical sensors (sensor fusion), the field-of-view for a given (microwave) imaging problem can be constrained to a volume bounded by the surface of the imaged object^32^. It is reported in^38^ that adopting such a prior constraint on the imaging problem can reduce the number of voxels to be reconstructed, and hence the complexity of the imaging problem, by more than 90%. Another approach includes the parallelization of the image reconstruction algorithm using general-purpose graphics processing units (GPGPUs)^39^ and field-programmable-gate-array (FPGA) structures^4^. However, despite the significant reduction in the size of the computational problem enabled by applying the aforementioned techniques, the reconstruction time can be on the order of several seconds^32^.

In view of this, it is evident that additional techniques are needed to further reduce the computational complexity of the imaging problem for real-time operation. Inspired by well-known lossy compression schemes such as JPEG or MP3, we hypothesize that a moderate loss of information can be tolerated in order to substantially reduce the memory and processing burden. Principal component analysis (PCA) is a mathematical tool that can be used in this perspective to decompose a matrix on a set of orthogonal bases^40^. In the considered imaging problem, the sensing matrix links the space to be imaged to the measured signals; by decomposing the sensing matrix into its principle components, we can identify independent structures of which the sensing matrix is composed and their respective contribution levels. We then select only the most significant principal components in order to compute a model of the sensing matrix with reduced dimensions.

Our proposal is distinct from recent ideas in the literature to optimize microwave imaging systems using PCA. One such approach, proposed in^41^ and^42^, is based on the radiation of illumination patterns specific to each type of scene to be imaged, necessarily requiring the use of reconfigurable systems for scene-dependent beam-synthesis. Provided a prior knowledge about the nature of the scene to be imaged is available, these authors have shown that it is possible to limit the number of sequential captures necessary for image reconstruction compared to the use of purely random patterns. Recent work in^43^ took this idea even further by directly integrating a model of the physical layer into an artificial neural network in order to jointly learn optimal measurement and processing strategies based on a priori knowledge of scene, task and measurement constraints. Since this "learned sensing" strategy enables one to minimize the acquisition of task-irrelevant information, it is highly task-specific and requires a supervised learning technique. In contrast to these prior works, the approach we propose here applies PCA to the sensing matrix rather than the expected scene. Consequently, our approach is not scene dependent and does not require the use of sequential measurements relying on active reconfigurable antennas. We propose a system-dependent but scene-independent method relying on a frequency-sweep to generate a succession of random illumination patterns that interrogate the scene to be imaged; via PCA we can limit the dimensionality of the sensing matrix and thereby the computational complexity of the image reconstruction.

This paper demonstrates that our approach makes it possible to reduce computation times and memory consumption for imaging applications while having only a minor impact on the quality of the reconstructed images. Our approach allows each application to dispose of a new degree of freedom in image reconstruction by moving the constraints to either image quality or numerical performance. The outline of this paper is as follows: In “Operation principle” section, we explain the concept of frequency-diverse computational microwave imaging and the application of PCA to the sensing matrix. In “Results and discussion” section, we present the images of a human-sized object reconstructed with and without PCA-based dimensionality reduction; moreover, we provide a quantitative analysis of the reconstructed images in terms of imaging quality, mean-square-error (MSE), memory consumption, and reconstruction time. Finally, we provide concluding remarks in the last section.

## Operation principle

### Frequency-diverse computational microwave imaging

In frequency-diverse computational imaging, a set of frequency-dependent spatially-varying field patterns are used to interrogate the scene to be imaged. Using the first Born approximation, the measured signal is linked to the imaged object by means of the sensing matrix (or the transfer function) of the imaging system as follows:

1 $$\begin{aligned} g_{i,j} (\omega ) = \int _{\varvec{r}} E_{Tx}^i(\varvec{r},\omega ) E_{Rx}^j(\varvec{r},\omega ) \rho (\varvec{r}) d\varvec{r} + n(\omega ). \end{aligned}$$

**Figure 1:**
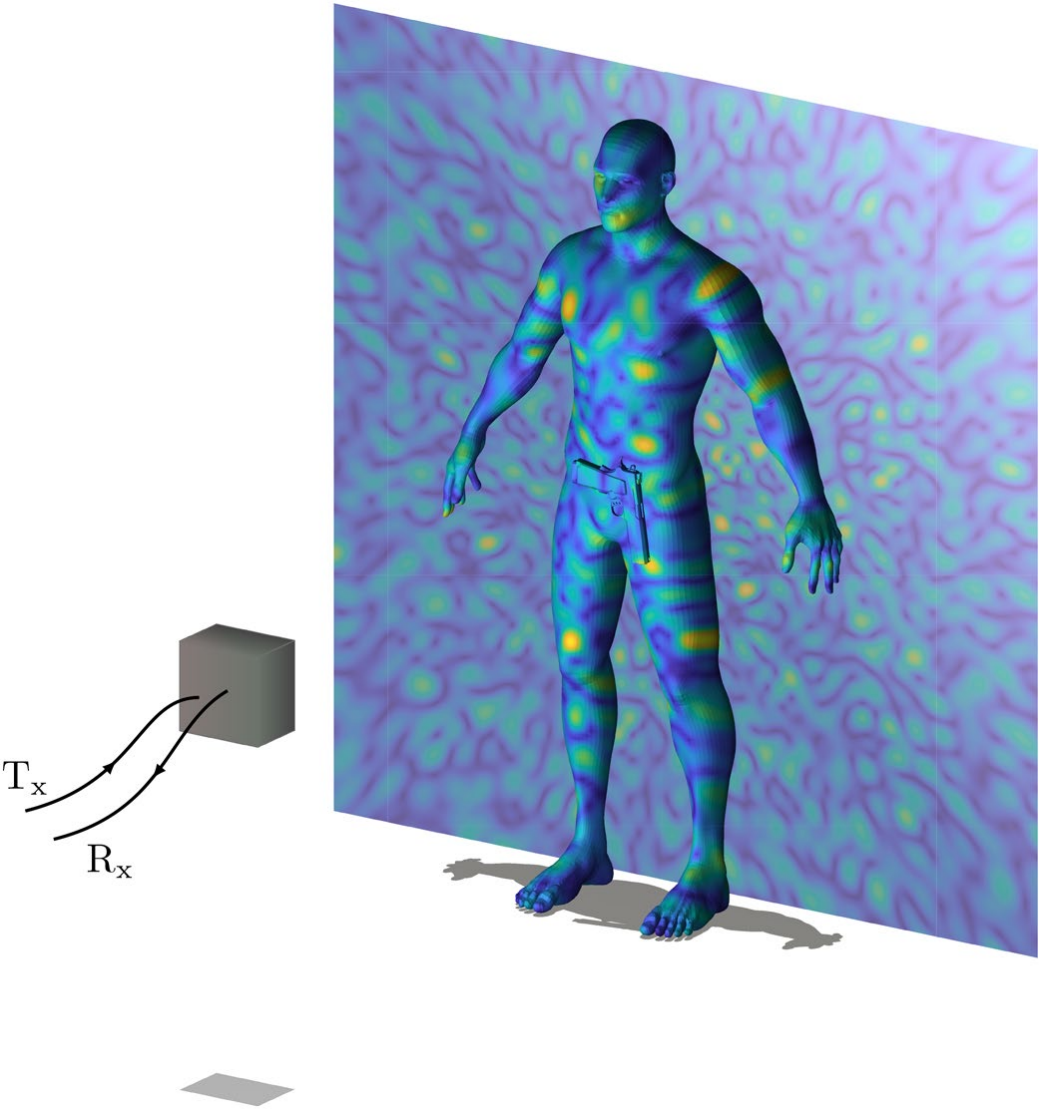
Illustration of a computational imaging system. The fields radiated in transmission and reception by a frequency diverse system interact with a target to be imaged. The scene ${\varvec{\rho }}$ is interrogated by a succession of quasi-random field distributions varying according to the frequency and measurement ports. By means of simple frequency sweeps, spatial information is quickly encoded into a set of measurements gathered in ${\mathbf {g}}$.

Equation (1) is the integral form of the forward model where *g* denotes the measurement signal, $\rho$ is the imaged scene, *i* and *j* are the indices of the transmit and receive frequency-diverse antennas, and $E_{Tx}$ and $E_{Rx}$ are the radiated *E*-field patterns of the transmit and receive antennas propagated to the scene using the free-space Green’s function^44^. Finally, $n(\omega )$ represents additive Gaussian white noise. For the sake of clarity, the principle of dimensionality reduction proposed in this paper is implemented within the framework of a scalar wave approximation. An adaptation of the studied techniques can nevertheless be carried out to the reconstruction of complete susceptibility tensors by means of recent developments in polarimetric computational imaging^33,45^. Under the first Born approximation, the sensing matrix is proportional to the dot product of the transmit and receive frequency-diverse antenna fields: $H \propto E_{Tx} E_ {Rx}$ (Fig. 1).

Retrieving an estimate of the reflectivity of the scene $\hat{\varvec{\rho }}$ is an inverse problem that is solved numerically based on a discretized version of the forward model (1). For a frequency-diverse aperture consisting of *T* transmit antennas and *R* receive antennas, and sampling the frequency band at *F* frequency points, the total number of measurement modes is $M = T \times R \times F$. The discretization of the scene is performed at the resolution limit of the aperture in the range and cross-range planes. The range resolution is governed by the imaging bandwidth $B=9$ GHz in the K-band ($17.5{-}26.5$ GHz): $\delta _r=c/2B=1.67$ cm; the cross-range resolution is governed by the aperture size *D*, target distance *d*, and wavelength $\lambda$: $\delta _{cr}= \lambda d/D$, resulting in $\delta _{cr}=7$ mm cross-range resolution for imaging at $d=1$ m distance. The $M \times N$ sensing matrix ${\mathbf {H}}$ links the *N* voxels to the *M* measurement modes. The discretized forward model is the following:

2 $$\begin{aligned} {\mathbf {g}}_{M \times 1} = {\mathbf {H}}_{M \times N}\, \varvec{\rho }_{N\times 1} + {\mathbf {n}}_{M\times 1}. \end{aligned}$$

In (2), although the field definitions forming the sensing matrix are scalar, bold font is used to denote the vector and tensor quantities. The sensing matrix ${\mathbf {H}}$ does not have an exact inverse because it is not square ($M \ne N$) and full rank. Several techniques can be used to retrieve an estimate of the scene information. Among those, direct reconstruction techniques can retrieve the scene estimate without the need for additional iteration steps as follows:

3 $$\begin{aligned} \hat{\varvec{\rho }} = {\mathbf {H}}^+ {\mathbf {g}} = {\mathbf {H}}^+ ({\mathbf {H}} {\varvec{\rho }}+{\mathbf {n}}) \approx {\varvec{\rho }}, \end{aligned}$$

where ${\mathbf {H}}^+$ denotes the reconstruction operator applied to the measurements in order to retrieve $\hat{\varvec{\rho }}$.

The simplest techniques, known as matched filtering, consists in using the conjugate transpose of the sensing matrix, denoted ${\mathbf {H}}^\dagger$, as reconstruction operator.

### Dimensionality reduction with principal component analysis

As noted above, for imaging problems with large scenes, such as in security screening, it can be challenging to retrieve the estimate of the scene in a timely manner due to the large size of the sensing matrix. In fact, processing a big sensing matrix might not even be possible due to a lack of available computational resources. For instance, in security-screening applications at K-band frequencies, the size of the sensing matrix without the use of any a priori knowledge was shown to be as large as 90 Gb^38^. Therefore, it is evident that additional techniques are needed to solve the inverse problem of Eq. (2) to retrieve $\hat{\varvec{\rho }}$, particularly for real-time applications.

The first step we take to lower the reconstruction problem’s computational complexity is the technique at the heart of the present paper: we use PCA to remove insignificant dimensions of the sensing matrix ${\mathbf {H}}$. PCA originates from the general context of spectral theory: ${\mathbf {H}}$ can be diagonalized using the singular value decomposition (SVD) technique^46^ that factorizes it into three sub-matrices,

4 $$\begin{aligned} {\mathbf {H}} = {\mathbf {U}} \varvec{{\Sigma }} \mathbf {V^\dagger }. \end{aligned}$$

**Figure 2:**
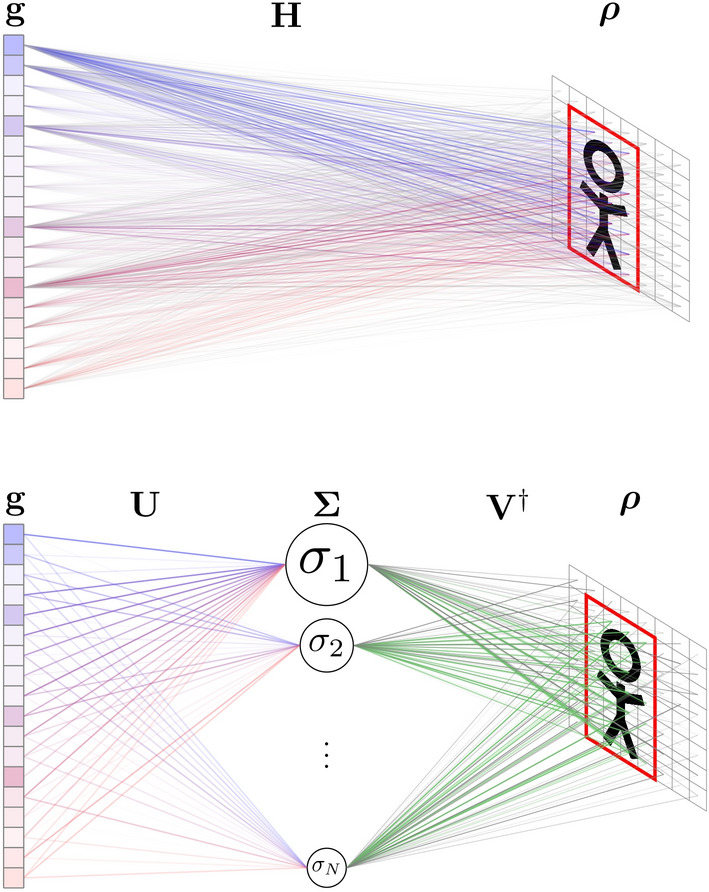
Top: Schematic representation of a measurement performed by a computational imaging system. The scene to be imaged $\varvec{\rho }$ is multiplied by a pre-determined sensing matrix ${\mathbf {H}}$. Linear combinations specific to the imaging system encode the spatial information into a set of measurements gathered in ${\mathbf {g}}$. Using information from optical sensors (sensor fusion), the model can be reduced on the fly to select only a portion of the region of interest, represented by the red rectangle. Bottom: The SVD allows one to have a new perspective on this interaction. Each column of ${\mathbf {V}}$ represents a spatial mask whose degree of similarity to the scene ${{\varvec{\rho }}}$ is estimated by correlation. Each of these masks is multiplied by a singular value that makes it possible to determine their level of contribution to the measured vector ${\mathbf {g}}$, through linear combinations of columns of ${\mathbf {U}}$. Once again, sensor fusion makes it possible to reduce the dimensions of the ${\mathbf {V}}$ matrix by selecting on the fly only useful portions of the region of interest.

In (4), ${\mathbf {U}}$ and ${\mathbf {V}}$ are orthonormal matrices and $\varvec{{\Sigma }}$ is a diagonal matrix whose non-negative coefficients $\sigma _n$ are organized by convention in descending order. An intuitive illustration of this decomposition is given in Fig. 2. The distribution of singular values (also referred to as singular value spectrum) directly reflects the degree of correlation between the rows of the sensing matrix^47^. An ideal imaging system will ensure that each measurement is a new (completely independent) source of information and that the sampling of the reconstructed image perfectly matches the resolution limit of this system. Under such ideal conditions, the SVD of the sensing matrix yields a decomposition with singular values of identical levels^48^, reflecting that ${\mathbf {H}}$ carries an optimal amount of information in relation to its dimensions. In practice, space and frequency dimensions are often oversampled in order to ensure a certain immunity to measurement uncertainties and additive noise. SVD is often used to study and improve the conditioning in inverse problems, limiting the amplification of subspaces that are weakly contributing and particularly sensitive to the impact of additive noise^30,49^.

Since the number of reconstructed voxels *N* is lower than the number of measurement modes *M*, there are *N* non-zero singular values $\sigma _n$ of which $K<N$ are deemed significant. The interaction with the scene to be imaged can thus be approximated as follows^50^:

5 $$\begin{aligned} {\mathbf {g}}&= {\mathbf {U}} \varvec{{\Sigma }} {\mathbf {V}}^\dagger {{\varvec{\rho }}} + {\mathbf {n}}\end{aligned}$$

6 $$\begin{aligned}&= \sum _{n=1}^{N} \sigma _n {\mathbf {u}}_n {\mathbf {v}}_n^\dagger {{\varvec{\rho }}} + {\mathbf {n}}\end{aligned}$$

7 $$\begin{aligned} {\mathbf {g}}&\approx \sum _{n=1}^{K} \sigma _n {\mathbf {u}}_n {\mathbf {v}}_n^\dagger \, {{\varvec{\rho }}}, \end{aligned}$$

where the measurement is approximated in the last step by a succession of $K<N$ orthogonal masks applied to the scene, neglecting the contribution of the lowest singular values and their associated singular vectors ${\mathbf {u}}_n$ and ${\mathbf {v}}_n^\dagger$. Rather than interrogating *N* voxels of the scene, it is then possible to try to reconstruct only *K* coefficients corresponding to spatial correlations with the masks formed by the most significant singular vectors ${\mathbf {v}}_n^\dagger$.

We now define a new matrix formalism of the imaging problem by selecting only the first *K* singular vectors, forming ${\mathbf {U}}_s$ and ${\mathbf {V}}_s$, and by creating a new diagonal matrix $\varvec{{\Sigma }}_s$ with the *K* most significant singular values. The measurement then takes the following form, revealing the principal components $\mathbf {P_c} = \mathbf {U_s} \varvec{{\Sigma }}_\mathbf{s }$ in the form of an operator interrogating ${\varvec{\alpha }} = {\mathbf {V}}_s^\dagger {{\varvec{\rho }}}$, a vector of *K* coefficients corresponding to independent linear combinations of the information of the scene to be imaged:

8 $$\begin{aligned} {\mathbf {g}}&\approx {\mathbf {U}}_\mathbf{s } \varvec{{\Sigma }}_\mathbf{s } \mathbf {V_s}^\dagger {{\varvec{\rho }}}\end{aligned}$$

9 $$\begin{aligned}&\approx \mathbf {P_c} {\varvec{\alpha }}. \end{aligned}$$

The matrix $\mathbf {P_c}$ has reduced dimensions $M \times K$ and inversion strategies similar to the ones already described for ${\mathbf {H}}$ can be implemented to obtain an estimate of the vector $\hat{\varvec{\alpha }}$ as follows:

10 $$\begin{aligned} \hat{\varvec{\alpha }} = \mathbf {P_c}^+ {\mathbf {g}}. \end{aligned}$$

Finally, taking advantage of the orthonormal properties of the ${\mathbf {V}}_\mathbf{s }$ matrix $(\langle {{\mathbf {V}}_i|{\mathbf {V}}_j}\rangle = \delta _{ij})$, the scene estimation is straightforward:

11 $$\begin{aligned} \hat{\varvec{\rho }}&= {\mathbf {V}}_\mathbf{s } \hat{\varvec{\alpha }} \end{aligned}$$

12 $$\begin{aligned}&\approx \mathbf {V_s} \mathbf {V_s}^\dagger {{\varvec{\rho }}} \end{aligned}$$

13 $$\begin{aligned} \hat{\varvec{\rho }}&\approx {{\varvec{\rho }}}. \end{aligned}$$

Rather than storing the matrix ${\mathbf {H}}$ of dimensions $M\times N$ for the application of iterative techniques or its reconstruction operator ${\mathbf {H}}^+$ for direct estimations, it is thus possible to store only two smaller matrices $\mathbf {P_c}$ (dimensions: $M \times K$) and $\mathbf {V_s}$ (dimensions: $N \times K$) or their respective reconstruction operators.

Having detailed the PCA-based dimensionality reduction, we now discuss how it can be combined with a priori knowledge from optical sensors to reduce the number of voxels to be reconstructed. Prior work^32,38,39,51^ has shown that based on optical sensors the surface boundaries of the object under consideration can be detected to define a volume around this surface that constrains the domain for microwave imaging (Fig. 2). Thereby, the number of voxels constituting the scene is reduced via support detection to $N_o<N$ elements, determining a new formalism valid for a given position of the target:

14 $$\begin{aligned} {\mathbf {g}}_{M \times 1} = \mathbf {H_o}_{M \times N_o}\, {\varvec{\rho }_o}_{N_o\times 1} + {\mathbf {n}}_{M\times 1}. \end{aligned}$$

It should be carefully noted that, as shown in Fig. 2, the pixel/voxel set $N_o$ selected based on information from optical sensors depends directly on the target position and must be continuously updated from the initial set of *N* elements defining the entire imageable scene.

Only the ${\mathbf {V}}_\mathbf{s }$ matrix is impacted by this support detection, reducing the number of reconstructed pixels from *N* to $N_o$. One can then estimate $\hat{\varvec{\rho }}_o$ in this reduced volume as follows:

15 $$\begin{aligned} \hat{\varvec{\rho }}_o&= {\mathbf {V}}_\mathbf{so} \hat{\varvec{\alpha }}. \end{aligned}$$

Our approach not only greatly reduces the memory consumption of an imaging system, but it also accelerates image reconstruction by reducing the number of required computational operations. We note that our approach remains fully compatible with the use of a priori knowledge from optical sensors which allows one to reduce the reconstructed domain in real time. In the following section, we validate our technique by studying a body scanner in a context oriented toward the detection of concealed threats.

## Results and discussion

### Considered imaging setup

**Figure 3:**
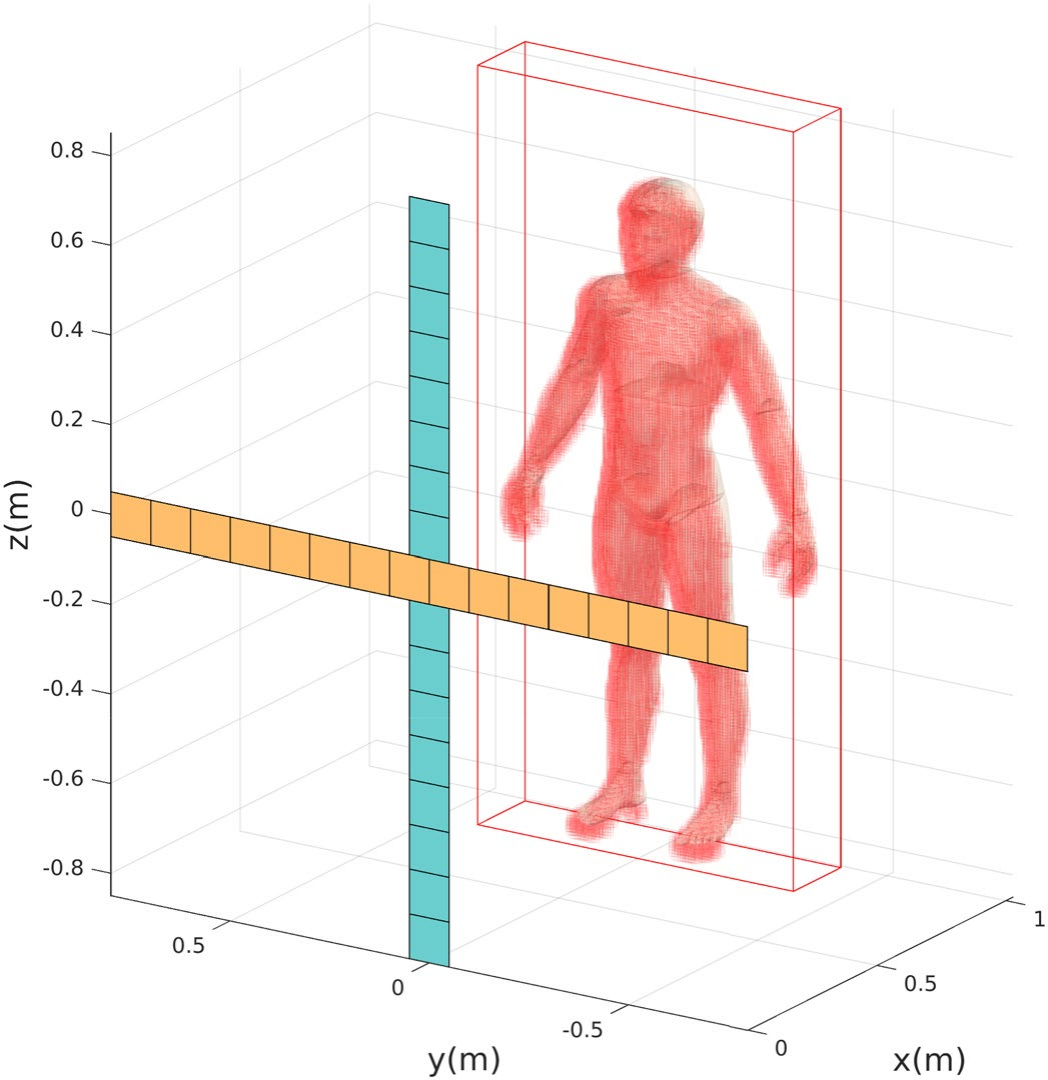
Synthesized composite frequency-diverse Mills-Cross aperture for imaging a human-sized object in the scene. The 16 metasurface antennas used in transmission (reception) are shown in orange (blue). The rectangular-shaped red volumetric frame represents the complete domain imaged by the ${\mathbf {H}}$-matrix interrogating a set of $N = 355{,}416$ voxels. Based on a priori knowledge, $N_o = 16{,}292$ are retained (depicted by red cubes forming the human-sized object) in a defined volume encompassing the front surface of the target.

Figure 3 shows the imaging scenario that we consider in simulation. This imaging system consists of 16 transmit and 16 receive metasurface antennas ($10\times 10 \text { cm}^2$), arranged in a Mills-Cross system layout. The latter represents a thinned array architecture while ensuring a similar *k*-space support extent in comparison to populating the entire aperture^31,52^. A human-sized object is placed at a distance of $d=1$ m from the aperture. The frequency-diverse imager operates at K-band frequencies (17.5–26.5 GHz), sampling the 9 GHz bandwidth at 101 frequency points which corresponds to a frequency interval of 90 MHz. We choose to work with 101 frequency sampling points based on the upper bound on the number of useful measurement modes that a frequency-diverse antenna with a finite quality (*Q*) factor can provide^36^, which is given by $QB/f_c$; for our system with antenna quality factor $Q=330$, bandwidth $B = 9$ GHz and central operating frequency $f_c=22$ GHz, the resulting upper bound is 135 modes. The synthesized frequency-diverse aperture thus produces $M = 25{,}856$ measurement modes. The initial region of interest contains $N = 12 \times 118 \times 251 = 355{,}416$ voxels; based on a priori knowledge from optical sensors, we narrow the number of voxels to be reconstructed down to $N_o = 16{,}292$.

**Figure 4:**
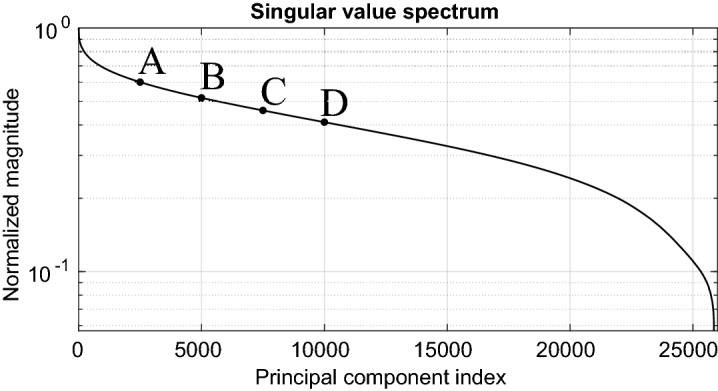
Singular value spectrum of the full ${\mathbf {H}}$-matrix. Imaging results are presented in this section by selecting four subsets of principal components, represented by cases A, B, C, and D, corresponding to the 2500, 5000, 7500, and 10,000 largest singular values, respectively.

The gains achievable in numerical performance by applying the dimensionality reduction of the sensing matrix that we propose inherently depend on the correlations between the utilized measurement modes. The considered imaging hardware has been optimized to generate a quasi-random set of measurement modes, meaning that an effort was made to keep the correlations low. This is evidenced by the singular value spectrum of the sensing matrix shown in Fig. 4 which is relatively flat. Therefore, gains in numerical performance achievable via dimensionality reduction of the sensing matrix are somewhat limited; nonetheless, even under these (for our proposal unfavourable) conditions, it is possible to illustrate the benefits of the proposed method. We anticipate that systems with lower performance and consequently a higher level of correlation between each measurement may benefit more strongly from a greater model reduction with a minor impact on the quality of the reconstructed images. The reconstructions in the following are performed on a computer with 128 Gb of RAM and an Intel Xeon processor with 10 cores running at 2.4 GHz (E5-2640 v4). To provide characteristic computation times that do not directly depend on the specifications of the utilized machine, we calculate ratios with respect to reference computation times evaluated with the full sensing matrix ${\mathbf {H}}$.

In the following, two data processing scenarios are studied to illustrate the impact of the proposed technique on the quality of the reconstructed images and on the associated computation times. First, we consider a matched filter as example of a direct reconstruction technique; second, we consider the generalized minimal residual method (GMRES) as example of an iterative reconstruction technique.

### Case 1: direct reconstruction by matched filtering

The first approach using a matched filter is based on a simple phase compensation of the sensing matrix. In the absence of any dimensionality reduction, image reconstruction by matched filtering takes the following form:

16 $$\begin{aligned} \hat{\varvec{\rho }}_{(\text {MF})} = {\mathbf {H}}^\dagger {\mathbf {g}}. \end{aligned}$$

**Figure 5:**
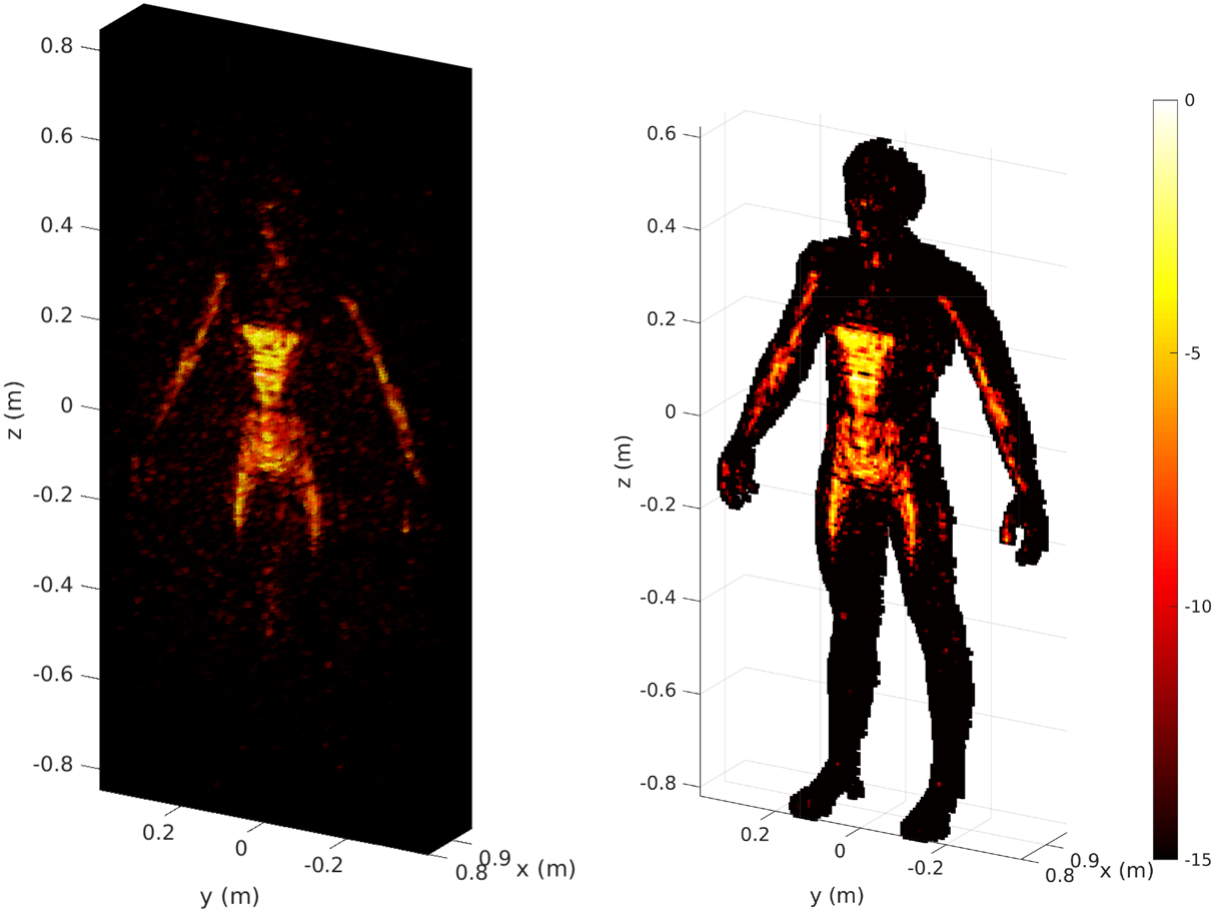
Reference scene reconstructions with matched filtering without the proposed PCA-based dimensionality reduction of the sensing matrix. Left: Scene estimate $\hat{\varvec{\rho }}$ computed for the entire region of interest ($N = 355{,}416$ voxels). Right: Scene estimate $\hat{\varvec{\rho }}_{o}$ computed only for the voxels selected from optical sensors ($N_o = 16{,}292$ voxels). The colormap is given in decibels with normalized data.

For reference, we show reconstruction results with matched filtering without the proposed PCA-based dimensionality reduction in Fig. 5, illustrating the difference between reconstructing all *N* voxels or only reconstructing the selected subset of $N_o$ voxels.

Matched filtering not only has the advantage of reducing the dimensions of the matrices manipulated for image reconstruction (in comparison to other techniques), but also of limiting the impact of clutter related to imperfect estimations outside of the reduced region of interest. Using this first simple reconstruction method as a benchmark, the advantages and disadvantages associated with PCA-based dimensionality reduction are now investigated.

**Figure 6:**
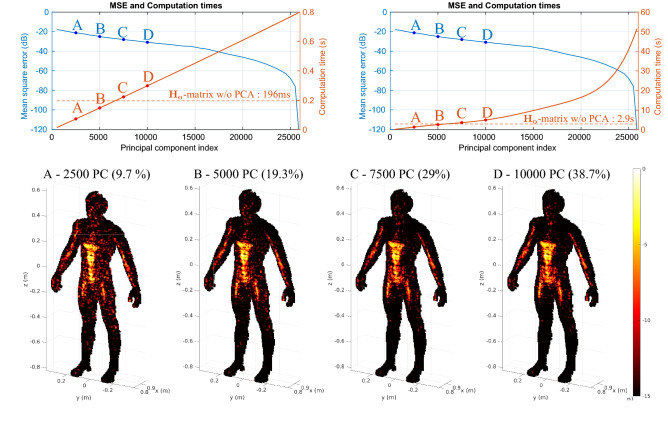
Study of the impact of dimensional reductions by PCA for direct reconstructions by matched filtering. The upper left graph corresponds to the raw image computation time and the upper right one includes, in addition to the computation time, the time required to extract the rows corresponding to the reduced region of interest from the $\mathbf {V_s}$-matrix according to the target position. The MSE is calculated with each reconstructed spatial reflectivity by considering the reconstruction with $\mathbf {H_o}$ as benchmark. The averaged computation times are represented for each reconstruction and the general trend is highlighted by means of a regression. The dashed red line represents the reconstruction time using the $\mathbf {H_o}^\dagger$ matrix in both cases. An animation presenting the successive reconstructions carried out by gradually increasing the number of principal components is available in the Supplemental Materials: https://bit.ly/3a7GFFy.

A selection of four reconstruction examples with matched filtering is presented in Fig. 6, computed following Eqs. (10) and (11). Reconstructions are carried out by calculating $\hat{\varvec{\rho }}_{o(\text {PCA,MF})} = {\mathbf {V}}_\mathbf{so} \, \mathbf {P_c}^\dagger {\mathbf {g}}$. We vary the number of principal components (PC) in steps of 500. In each case, the $\mathbf {P_c}$ and $\mathbf {V_{s}}$ matrices can be pre-calculated and stored in memory to be used on different measurements ${\mathbf {g}}$; this reduces the computational burden during operation since for a given scenario it is not necessary to change the number of selected principle components. The reconstructed images are then compared to the reconstructions achieved with $\mathbf {H_o}^\dagger$. It is therefore intuitive that beyond a certain number of principal components, the intermediate factorization step ultimately requires more calculation than for a unique multiplication by the conjugate-transpose of the sensing matrix. This phenomenon is seen in Fig. 6 when more than 5700 PCs (22%) are selected, leading to an average computation time overcoming the 196 ms threshold required to evaluate Eq. (16).

Example B in Fig. 6 requires a reconstruction time slightly below that of the reference case; upon visual inspection, the quality of the reconstructed image appears to be comparable to cases C and D which only use 29% and 38.7% of the principal components. We evaluate the MSE by taking the reconstruction with the $\mathbf {H_o}$ matrix as reference in order to assess the loss in image quality. Indeed, the gradient of the MSE curve seen in Fig. 6 is very low at the beginning. Thus, by moderately sacrificing the accuracy of the reconstructions it is possible to improve the reconstruction speed (and hence refresh rate) with our proposed approach.

**Figure 7:**
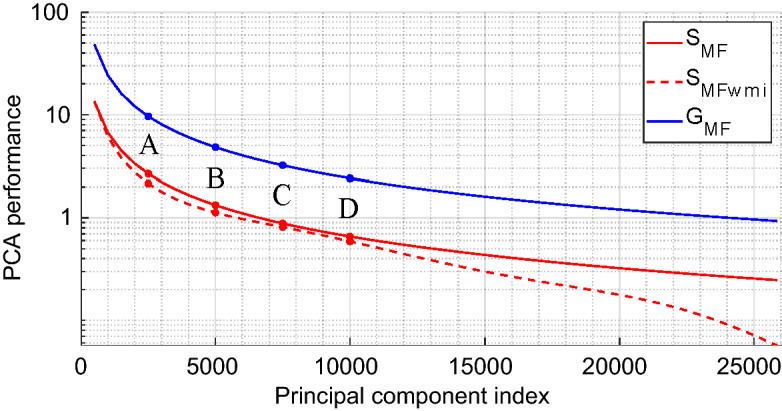
Reduction of the computational burden through PCA-based dimensionality reduction of the sensing matrix in direct reconstructions (matched filtering). We plot the reduction in computation time ($S_\text {MF}$, red) and memory usage ($G_\text {MF}$, blue). The red dashed curve denoted by $S_{\text {MFwmi}}$ additionally accounts for the impact of ${\mathbf {H}}$ and ${\mathbf {V}}_\mathbf{s }$ matrix indexing times (wmi stands for “with matrix indexing”).

An analysis of the achieved reduction of the computational burden for the considered matched-filtering technique is shown in Fig. 7 as a function of the number of retained principal components. The $S_{\text {MF}}$ curve corresponds to the ratio between the raw image-computation time using PCA-based dimensionality reduction of the sensing matrix relative to that using the full $\mathbf {H_o}$-matrix. We also plot a second curve, $S_{\text {MFwmi}}$, that in addition to the raw computation times accounts for the time it takes to extract the $\mathbf {H_o}$ or $\mathbf {V_{so}}$ sub-matrices from ${\mathbf {H}}$ or $\mathbf {V_s}$, respectively. As seen in the figure, for the four considered truncations A to D, the gains in computation time are comparable with and without accounting for the time that the matrix-indexing takes. Matrix-indexing only begins to adversely affect the latency gain once a very large number of singular values is selected. Yet, as seen in Fig. 6, the absolute extraction times of the sub-matrices in our study for each new position of the target in the region of interest are not compatible with real-time applications to a moving scene. The reason for this is that we performed our study on a CPU with MATLAB which suffers from notoriously poor matrix-indexing efficiency. However, space-partitioning strategies associated with the use of GPUs and low-level programming can achieve a latency compatible with real-time applications^32^.

For direct reconstruction techniques like matched filtering, the PCA-based dimensionality reduction also yields a much more direct benefit related to memory usage. Real-time applications require the ability to store all matrices needed for computations in a fast memory; body-scanning applications are then particularly constrained by the volume of data used to calculate each image. The memory saving factor is defined by the following equation:

17 $$\begin{aligned} G_{\text {MF}} = \frac{\text {size}({\mathbf {H}})}{\text {size}(\mathbf {P_c}) + \text {size}({\mathbf {V}}_\mathbf{s })} = \frac{M \times N}{(M+N) \times K}. \end{aligned}$$

As a reminder, *M* corresponds to the number of utilized measurement modes, *N* to the total number of voxels and *K* to the number of selected principal components. We emphasizee that the total number of voxels *N* is taken into account in Eq. (17) so as to not restrict the study to a target specific sub-domain. Based on Eq. (17), we can determine a limiting value of *K* beyond which the memory consumption will exceed the one in case of using the entire sensing matrix, that is when $G_{\text {MF}}<1$. The latter happens for $K > M \times N / (M + N) = 24{,}102$ in the considered imaging scenario, equivalent 93% of the total number of principle components in the studied case. We present the evolution of the memory saving as a function of the number of retained principle components in Fig. 7.

The application of our PCA-based dimensionality reduction enabled memory-consumption saving factors of 9.6, 4.8, 3.2 and 2.4 in the considered cases A, B, C, and D, respectively. We used complex values coded in simple precision on 8 bytes such that ${\mathbf {H}}$ occupies more than 68 Gb, which justifies the usefulness of the proposed approach. In conclusion of this first study on a direct reconstruction technique, we have evidenced the possibility to slightly accelerate the image reconstruction (on average about 148 ms vs 196 ms for the reference) while simultaneously significantly reducing the RAM consumption by a factor of 4.8—at the price of a minor image degradation that does not hamper potential security-screening applications.

### Case 2: iterative reconstruction with GMRES

Reconstructions by matched filtering have the advantage of being fast; however, they do not allow for the compensation of magnitude terms. More advanced iterative techniques that improve the reconstruction quality can be particularly slow, especially when the dimensions of the problems to be treated are large. The PCA-based dimensionality reduction is now applied to this new context of iterative techniques to study the possible savings in computation time and memory consumption. Specifically, the generalized minimal residual method (GMRES) is applied, having yielded good performances in previous work^30,32^. Since this technique requires the use of a square sensing matrix, the $\mathbf {P_c}^\dagger$ matrix is multiplied to each member of (8):

18 $$\begin{aligned} \mathbf {P_c}^\dagger {\mathbf {g}}&\approx \mathbf {P_c}^\dagger \mathbf {P_c} {\varvec{\alpha }}. \end{aligned}$$

**Figure 8:**
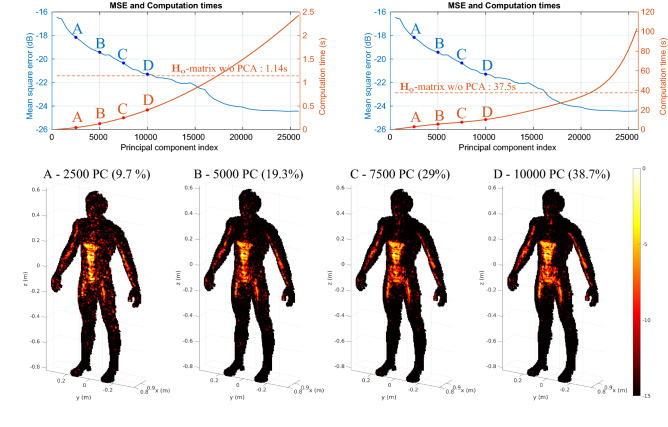
Study of the impact of dimensional reductions by PCA for iterative reconstructions by the general minimum residual method (GMRES). The upper left graph corresponds to the raw image computation time and the upper right one includes in addition the time required to extract the rows corresponding to the reduced region of interest from the $\mathbf {V_s}$-matrix according to the target position. The MSE is calculated with each reconstructed spatial reflectivity by considering the GMRES reconstruction with the matrix $\mathbf {H_o}$ as benchmark. The averaged computation times are represented for each reconstruction and the general trend is highlighted by means of a regression. The dashed red line represents the reconstruction time with the $\mathbf {H_o}$ matrix in both cases. An animation presenting the successive reconstructions carried out by gradually increasing the number of principal components is available in the Supplemental Materials: https://bit.ly/2PuiWYl.

By minimizing $||\mathbf {P_c}^\dagger {\mathbf {g}} - \mathbf {P_c}^\dagger \mathbf {P_c} {\varvec{\alpha }} ||_ 2$, this iterative approach enables the reconstruction of the vector $\hat{\varvec{\alpha }}$ starting from a matched-filtering estimate $\mathbf {P_c}^\dagger {\mathbf {g}}$ and with use of the correlation matrix of the principal components $\mathbf {P_c}^\dagger \mathbf {P_c}$. In a similar way to the first study, the performances are studied for a set of reconstructions carried out using different amounts of leading singular values. Four examples are more carefully investigated by displaying the reconstructed images (Fig. 8).

Compared to reconstructions performed only by matched filtering, magnitude-term compensation provides more accurate estimates of the imposed constant reflectivity function over the entire target. However, the specular nature of the interaction of waves with the target tends to create shadows that cannot be removed unless the target is illuminated from other incident angles. This physical limitation, also affecting conventional imaging systems, can nevertheless be mitigated by merging a set of reconstructions from different perspectives^32,53^.

As in the previous section, we plot the convergence of the reconstruction quality in terms of the MSE for the use of different amounts of leading principle components in Fig. 8. The result obtained with only 6.8% of the principal components (case A) makes it possible to distinguish the first main structures of the image but still remains corrupted by the lack of information. The addition of information to reach 22.7% of the principal components allows the reconstruction of a much more exploitable image revealing significantly more detail. Once again, it is difficult to discern upon visual inspection the difference between the reconstructions carried out with 43% and 100% of the principal components.

The effect of the PCA-based dimensionality reduction on the calculation time depends again on the number of selected principal components. Although the results obtained with 100% of the principal components are identical to those obtained by applying the same technique with the ${\mathbf {H}}$ matrix, the PCA-based approach still requires in this case an additional calculation step justifying a longer time beyond a certain number of principal components.

As before, we also compare the memory consumption with and without dimensionality reduction. For this purpose, the ratio of matrix sizes required to reconstruct images from frequency measurements alone is calculated for the iterative GMRES technique. If the full sensing matrix is used by GMRES, the quantity $||{\mathbf {H}}^\dagger {\mathbf {g}} - {\mathbf {H}}^\dagger {\mathbf {H}}\, {\varvec{\rho }} ||_ 2$ is minimized, which requires storage of both ${\mathbf {H}}$ and additionally the correlation matrix $\mathbf {H^\dagger H}$. The reduction in memory usage achieved by PCA-based dimensionality reduction is thus given by

19 $$\begin{aligned} G_{\text {GMRES}}&= \frac{\text {size}(\mathbf {H^\dagger H})+ \text {size}({\mathbf {H}})}{\text {size}(\mathbf {P_c}^\dagger \mathbf {P_c}) + \text {size}(\mathbf {P_c}) + \text {size}({\mathbf {V}}_\mathbf{s })}\end{aligned}$$

20 $$\begin{aligned}&= \frac{(N + M) \times N}{(K+M+N) \times K}. \end{aligned}$$

**Figure 9:**
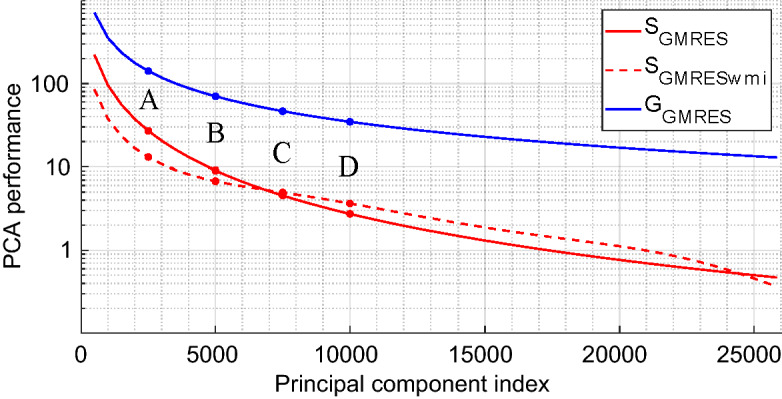
Reduction of the computational burden through PCA-based dimensionality reduction of the sensing matrix in iterative reconstructions (GMRES). We plot the reduction in computation time ($S_\text {GMRES}$, red) and memory usage ($G_\text {GMRES}$, blue). The red dashed curve denoted by $S_{\text {GMRESwmi}}$ additionally accounts for the impact of ${\mathbf {H}}$ and ${\mathbf {V}}_\mathbf{s }$ matrix indexing times (wmi stands for “with matrix indexing”).

Our dimensionality-reduction technique hence enables us to limit memory usage as long as $K<(\sqrt{M^2 + 5N^2 + 6MN} - (M+N))/2$, or 223,914 principal components in this study (impossible case here since we only dispose 25,856 singular values). The memory gain $G_{\text {GMRES}}$, which is thus always greater than 1, is plotted in Fig. 9.

Having considered a more computationally intensive image reconstruction based on an iterative technique in this section, the utility of the proposed approach was even more evident. For example C (see Figs. 8 and 9), the calculation time is divided by a factor of 3.5 and the RAM consumption by a factor of 46.5—yielding an image with a MSE of $-20.3$ dB. The lowest achievable MSE of $-24.4$ dB is only achieved if all principle components are used.

**Figure 10:**
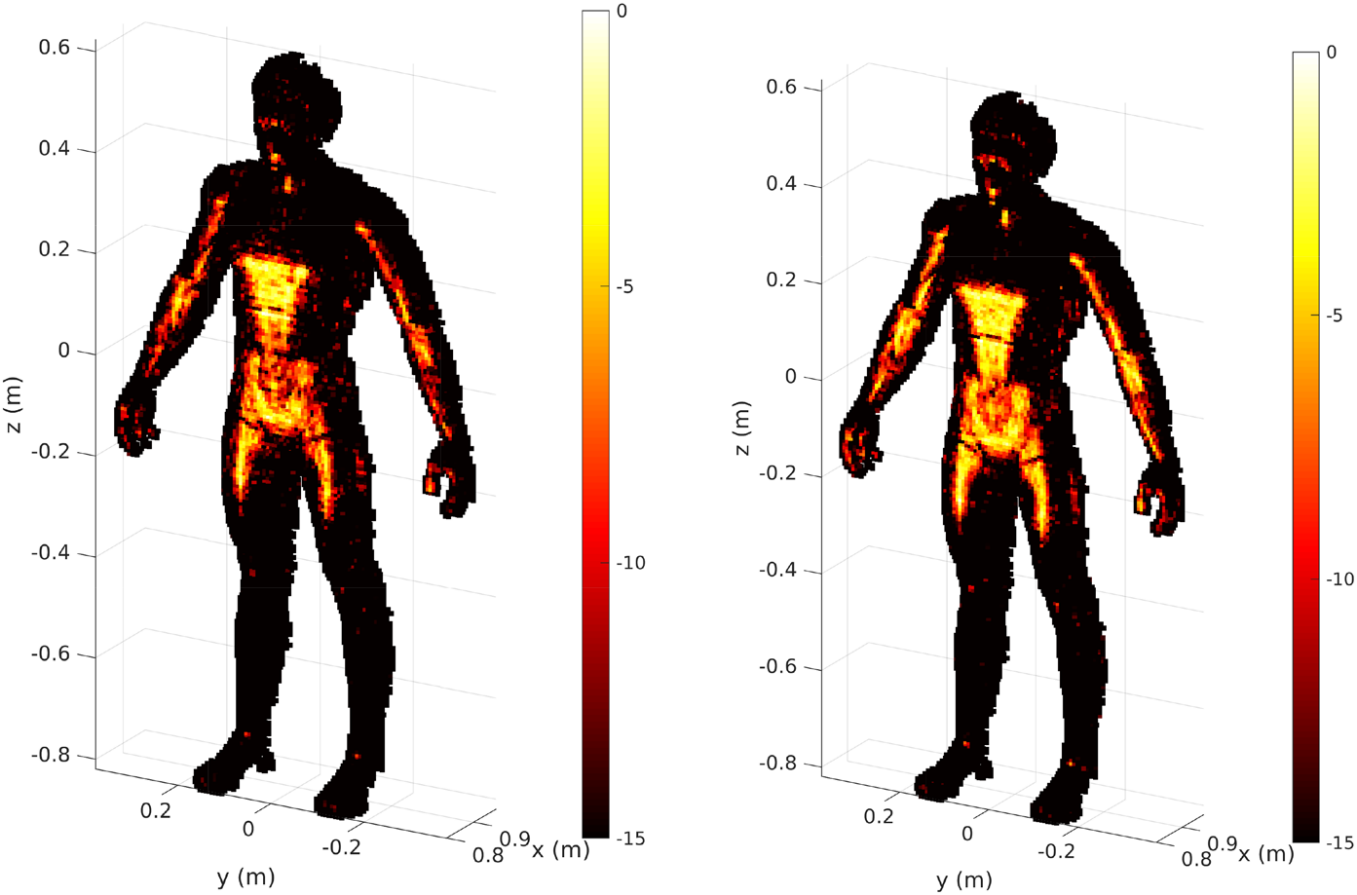
Comparison of GMRES-based reconstructions performed using the PCA-based technique with all principal components (left) and using the $\mathbf {H_o}$ matrix (right).

Finally, we use the GMRES results to illustrate the impact of the non-orthogonality of ${\mathbf {V}}_\mathbf{so}$. In order to avoid a dependence on the position of the target within the region of interest, the singular vectors ${\mathbf {V}}_\mathbf{so}$ are extracted from the singular vectors ${\mathbf {V}}_\mathbf{s }$ corresponding to the full sensing matrix ${\mathbf {H}}$ for the entire region of interest. By limiting the support of the reconstruction, the orthogonality of the ${\mathbf {V}}_\mathbf{so}$ sub-matrix is not guaranteed, especially since the number of reconstructed pixels may be greater than the number of selected principal components. This limitation is illustrated in Fig. 10 by comparing the result obtained with GMRES using the matrix $\mathbf {H_o}$ and the result obtained with GMRES using all principal components in the PCA-based approach. To mitigate the negative effect of this spatial filtering, one could compute the pseudo-inverse of ${\mathbf {V}}_\mathbf{so}$ for each new target position. However, this approach would require an additional calculation step for each reconstructed image, limiting the achievable refresh rate of the system under consideration.

### Effects of measurement noise

This study concludes with an analysis of the impact of thermal noise usually associated with the use of active receivers. Previous work has highlighted the usefulness of PCA in the identification^54^ and filtering^55^ of noise components of signals in various imaging applications such as X-ray tomography^56^ and magnetic resonance imaging^57^. The decomposition of linear operators by PCA facilitates the study of noise corruption, revealing its impact on the weakest principal components in a degree directly dependent on the signal-to-noise ratio (SNR). The elimination of these weakest components thus leads to the suppression of noise subspaces, implicitly realizing a regularization by truncated singular value decomposition^58^.

The previous sections have highlighted the beneficial effects of dimensionality reduction of sensing matrices on image computation times and memory consumption. As a continuation of these efforts, it is finally proposed to study the effects of additive measurement noise on the proposed PCA-based technique. Following the model given by Eq. (2), thermal noise is added to the measurements, following a Gaussian distribution of zero mean and standard deviation $\sigma _{n}$ such that ${\mathbf {n}} \sim {\mathcal {N}}(0,\sigma _n^2)$. The noise standard deviation is determined to reach an objective SNR from the measured signal power. It will be considered in the following that the sensing matrix can be perfectly determined and will thus be noise-free. This assumption seems reasonable insofar as this matrix is measured in a preliminary stage without any specific time constraint, opening the possibility of averaging measurements and of increasing integration time of each acquired sample.

**Figure 11:**
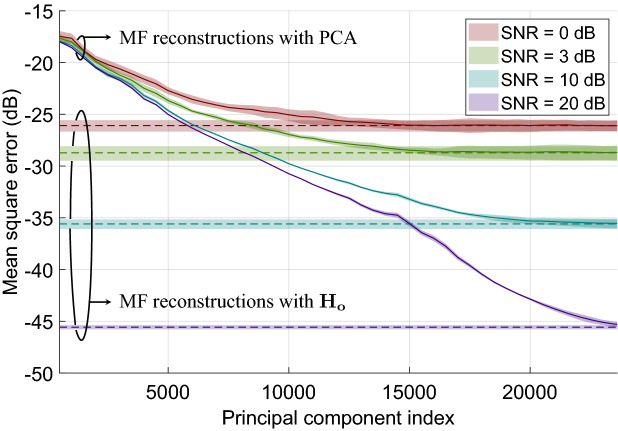
Evolution of the MSE of reconstructions computed by matched filtering (MF) in the presence of Gaussian additive noise. The MSE is calculated for each estimate with the noise-free reconstruction carried out with the $\mathbf {H_o}^\dagger$ matrix chosen as a reference. The continuous lines represent the reconstructions performed using the proposed PCA-based technique while the dotted lines correspond to the reconstructions with the $\mathbf {H_o}$ matrix. These results correspond to the means computed in each case with 10 simulations carried out with different noise realizations satisfying objective SNR, while the associated colored envelopes correspond to a variation of plus or minus one standard deviation around these means.

For a series of SNRs of noise-corrupted measured signals, a matched-filtering reconstruction is performed. The figure of merit is again defined as the MSE for each estimate, considering the noise-free reconstruction with $\mathbf {H_o}^\dagger$ as a reference. For the different considered SNRs, 10 measurements are simulated to compute the mean and standard deviation of the MSE in each case. Figure 11 displays the average and standard deviation of the MSE for four considered SNRs as a function of the number of retained principal components. For reference, the MSE achieved with the full sensing matrix $\mathbf {H_o}$ is also indicated.

As expected, the quality of the PCA-based reconstructions eventually converges to that of reconstructions based on the full sensing matrix $\mathbf {H_o}$ once a sufficient number of principal components is retained. The latter depends on the SNR of the measurements. Indeed, as the noise level increases, a growing number of subspaces are corrupted, reducing the overall number of uncorrupted subspaces in $\mathbf {H_o}$ that can contribute new information to the reconstruction. These results lead to the conclusion that the proposed PCA-based technique is particularly suitable for the reconstruction of images from measurements with unfavorable SNR. Under such conditions, PCA-based reconstructions can indeed be faster and less memory-intensive with little to no impact on the quality of the resulting images compared to results obtained from a full sensing matrix.

## Conclusion

Since computational imaging systems typically cannot ensure perfect spatial and spectral orthogonality of the utilized measurement modes, there is usually a non-negligible level of correlation between the obtained measurements. These correlations imply that upon a change of basis via singular value decomposition, many components of the sensing matrix contribute only weakly; neglecting such insignificant components thus entails only a minor deterioration of the imaging quality but offers significant reductions in memory usage and enhancements in computation speed. Although the suppression of the least contributing principal components generally results in a loss of quality of the reconstructed images, this study also highlighted that many of these suppressed components cannot contribute to the reconstruction under realistic operating conditions due to the presence of noise anyway. Our study illustrated the proposed dimensionality reduction of the sensing matrix with a case study of a specific computational imaging system based on a Mills-Cross aperture composed of metasurface antennas and a complex target (a human), considering both direct and iterative image reconstruction techniques. The finite spatial extent and intrinsic losses of the considered metasurface antennas result in notable correlations between the utilized measurement modes. Our results offer the possibility to trade-off memory needs and computation time with the image reconstruction quality, offering a new degree of freedom in computational imaging to place the emphasis on either reconstruction quality or numerical performance. Our method can readily be applied to other imaging systems and contexts that rely on sensing matrices with inevitable correlations^36,59^. Finally, we note that the reconstruction techniques used in this paper are agnostic to the scene to be imaged, thus imposing no restrictions on the nature or on the sparsity of the region of interest. Future efforts may investigate whether the presented dimensionality reduction technique can be combined with structure-aware imaging algorithms^41–43,60^.

## Supplementary Information

Supplementary Information 1.

Supplementary Information 2.

Supplementary Information 3.
