# Supplementary material for: Lowering latency and processing burden in computational imaging through dimensionality reduction of the sensing matrix
Source: Sci Rep. 2021 Feb 11;11:3545. doi: 10.1038/s41598-021-83021-6 (PMC7878915; doi:10.1038/s41598-021-83021-6)
Supplement: Supplementary file 1 — Supplementary Information 1. [file 41598_2021_83021_MOESM1_ESM.pdf]

# Lowering latency and processing burden in computational imaging through dimensionality reduction of the sensing matrix

**Thomas Fromentèze[1,*,+], Okan Yurduseven[2,+], Philipp del Hougne[3], and David R. Smith[4]**

[1]XLIM Research Institute, University of Limoges, Limoges, 87060, France
[2]Centre for Wireless Innovation (CWI), Institute of Electronics, Communications and Information Technology (ECIT), School of Electronics, Electrical Engineering and Computer Science (EEECS), Queen's University Belfast, Belfast, BT3 9DT, UK
[3]Institut de Physique de Nice, CNRS UMR 7010, Université Côte d'Azur, 06108 Nice, France
[4]Department of Electrical and Computer Engineering, Center for Metamaterials and Integrated Plasmonics,Duke University, Durham, NC 27708, USA
[*]thomas.fromenteze@unilim.fr
[+]these authors contributed equally to this work

## ABSTRACT

Recent demonstrations have shown that frequency-diverse computational imaging systems can greatly simplify conventional architectures developed for imaging by transferring constraints into the digital layer. Here, in order to limit the latency and processing burden involved in image reconstruction, we propose to truncate insignificant principal components of the sensing matrix that links the measurements to the scene to be imaged. In contrast to recent work using principle component analysis to synthesize scene illuminations, our generic approach is fully unsupervised and is applied directly to the sensing matrix. We impose no restrictions on the type of imageable scene, no training data is required, and no actively reconfigurable radiating apertures are employed. This paper paves the way to the constitution of a new degree of freedom in image reconstructions, allowing one to place the performance emphasis either on image quality or latency and computational burden. The application of such relaxations will be essential for widespread deployment of computational microwave and millimeter wave imagers in scenarios such as security screening. We show in this specific context that it is possible to reduce both the processing time and memory consumption with a minor impact on the quality of the reconstructed images.

This file includes:
Supplementary animations 1 and 2
These two GIF animations are made within the framework of the body scanner application studied in this paper.

## Supplementary animation 1:

`Animation-Matched_Filtering+PCA.gif`

This animation shows the evolution of the reconstructions carried out by Matched Filtering by selecting an increasing number of principal components.

## Supplementary animation 2:

`Animation-GMRES+PCA.gif`

This animation shows the evolution of the reconstructions carried out by Generalized Minimal Residual Method by selecting an increasing number of principal components.
