# Supplementary figures and images for: Lowering latency and processing burden in computational imaging through dimensionality reduction of the sensing matrix
Source: Sci Rep. 2021 Feb 11;11:3545. doi: 10.1038/s41598-021-83021-6 (PMC7878915; doi:10.1038/s41598-021-83021-6)

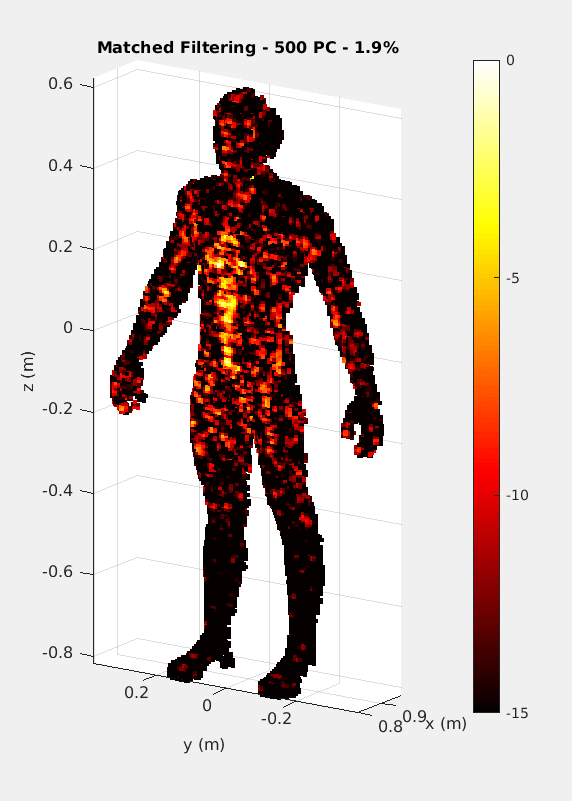

Supplement: Supplementary file 2 — Supplementary Information 2. [file 41598_2021_83021_MOESM2_ESM.gif]

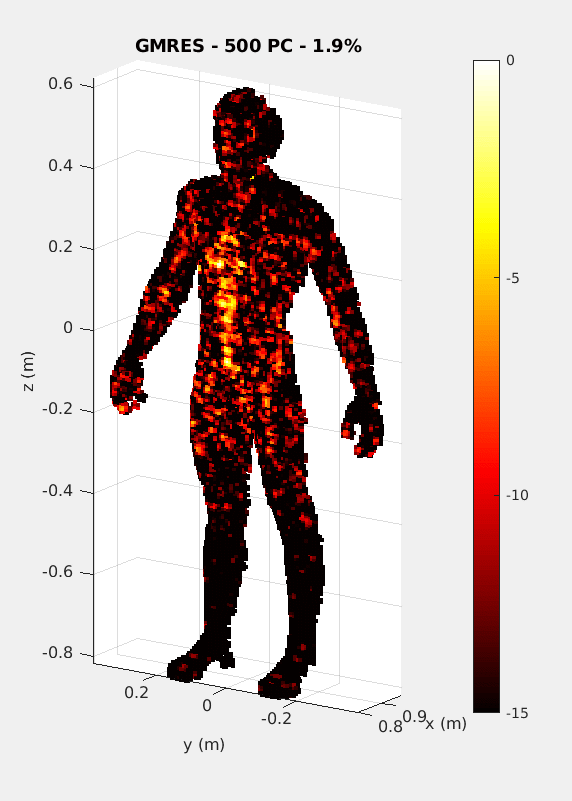

Supplement: Supplementary file 3 — Supplementary Information 3. [file 41598_2021_83021_MOESM3_ESM.gif]
